# Facile Preparation of Wormlike Graphitic Carbon Nitride for Photocatalytic Degradation of Ustiloxin A

**DOI:** 10.3390/nano10112256

**Published:** 2020-11-14

**Authors:** Yanfei Wu, Jin Mao, Chuanwei Ao, Di Sun, Xiaorui Wang, Qin Hu, Xuezhu Du, Feng Sheng

**Affiliations:** 1 State Key Laboratory of Biocatalysis and Enzyme Engineering, School of Life Sciences, Hubei University, Wuhan 430062, China; 201711110710913@stu.hubu.edu.cn (Y.W.); 201911110711057@stu.hubu.edu.cn (C.A.); 201911110711093@stu.hubu.edu.cn (X.W.); 20180136@hubu.edu.cn (Q.H.); 20080017@hubu.edu.cn (X.D.); 2 National Reference Laboratory for Agricultural Testing P.R. China, Key Laboratory of Detection for Mycotoxins, Laboratory of Quality & Safety Risk Assessment for Oilseed Products (Wuhan), Ministry of Agriculture, Oil Crops Research Institute, Chinese Academy of Agricultural Sciences, Wuhan 430062, China; 82101181101@caas.cn

**Keywords:** g-C_3_N_4_, wormlike, photocatalytic degradation, ustiloxin A

## Abstract

Natural toxic contaminants have been recognized as threats to human health. Ustiloxins are the toxic secondary metabolites of fungus generated from rice false smut disease, which are harmful to animal/human reproduction and growth. However, there are rare researches on the control and reduction of ustiloxins through physical, chemical and biological ways. Herein, we demonstrated that photocatalysis of semiconductor nanomaterials could be as a potential way to degrade or mitigate the contamination of ustiloxin A. A kind of wormlike graphitic carbon nitride (g-C_3_N_4_) was facilely prepared from modified dicyandiamide precursor via pyrolysis method and characterized by X-ray diffraction, high-resolution transmission electron microscope and X-ray photoelectron spectroscopy etc. It was found that g-C_3_N_4_ from modified dicyandiamide precursor showed better activity for ustiloxin A degradation under visible light irradiation than that of pristine g-C_3_N_4_. This was ascribed to the lager specific surface area, more uniform microstructure, better photogenerated charges separation and transformation of wormlike g-C_3_N_4_ compared with pristine g-C_3_N_4_. Most important, the structure of degradation intermediates and the possible pathway were proposed based on the results of high-performance liquid chromatography-mass spectrometry after 80 min photoreaction treatment. Our findings may provide a green, efficient way for ustiloxins mitigation and useful information for future study.

## 1. Introduction

**Figure 1:**
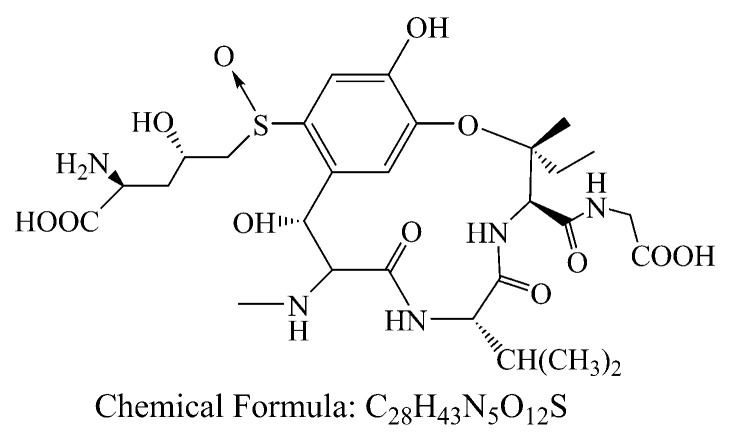
Chemical structures of ustiloxin A.

With the development of economy, society and technology, the environment pollutions become more and more serious. Besides antibiotics, heavy metal, dye and pigment from industrial activities, natural contaminants such as biotoxins and pathogenic microorganism with high toxicity should be also concerned in daily life. Ustiloxins are the secondary metabolites of fungus generated from rice false smut disease that is a worldwide fungal disease [1,2]. Ustiloxins can cause antimitotic behavior via preventing microtubule assembly and skeleton formation of eukaryotic cells, and seriously threaten animal/human reproduction and growth [3,4,5]. Among six identified ustiloxins, ustiloxin A (UA) and B are the main forms found with about 80 percent of the total ustiloxins in environment. In these days, most attentions were paid to study the occurrence, detection and biosynthesis of ustiloxins [6,7,8,9,10]. It was worth noting that the UA (C_28_H_43_N_5_O_12_S, 673.73, Figure 1) existed in the surface water from two paddy field, and the concentrations were 2.82 and 0.26 μg/L in Enshi, Hubei, China [8]. This indicated that this toxin could release into the environment, which might be as a potential threat to human and animals. However, there are few reports about how to reduce UA via chemical, physical and biological methods.

Recently, there is an incremental interest in photocatalytic technology of semiconductor nanomaterials applied to biotoxins mitigation [11,12,13,14,15,16]. Compared to chemical, physical and biological methods, photocatalytic technology has many superiorities: environment-friendly, low cost, mild reaction and large-scale application [11]. It was found that graphene/ZnO composites presented efficient activities to degrade deoxynivalenol under UV light irradiation, and the possible intermediates were proposed [12]. Microcystin-LR can be also degraded over magnetic N-doped TiO_2_ composite under visible light irradiation [13]. Our studies indicated that graphitic carbon nitride, WO_3_ and their composites had favorable abilities for degradation of aflatoxin B_1_ under visible light irradiation, and derived the possible degradation mechanism and pathway [14,15,16]. Based on these previous reports, it can be concluded that photocatalytic technology may be a potential way to mitigate and reduce the biotoxins. 

As a metal-free, visible light responsive and nontoxic semiconductor catalyst, graphitic carbon nitride (g-C_3_N_4_) with a suitable band gap of ca. 2.7 eV has attracted many researchers’ attentions [17,18,19,20]. However, the activities of pristine g-C_3_N_4_ was suppressed by lesser number of the active sites, high recombination of photogenerated charges and long transfer distance from inside to surface. Many efforts were performed to enhance the photocatalytic activities through morphological control [21], doping with metal [22] and non-metal and construction of heterojunction or Z-scheme composites [23,24]. The control and optimization of precursor for g-C_3_N_4_ synthesis has been regarded as a facile way to enhance its chemical and physical properties [25]. Different precursors and conditions can lead to as-prepared g-C_3_N_4_ with different microstructure and photoelectrical properties, which have been applied to degrade organic pollutants, hydrogen generation and CO_2_ reduction [26,27,28,29,30].

Herein, a kind of wormlike g-C_3_N_4_ was prepared by a simple one-step thermolysis method from modified dicyandiamide precursor. The modified precursor was from the recrystallization of dicyandiamide in centrifuge tubes without grinding process, which was calcined at 580 °C to obtain as-prepared wormlike g-C_3_N_4_. The wormlike g-C_3_N_4_ was characterized by X-ray Diffraction, high resolution transmission electron microscopy, X-ray photoelectron spectroscopy and UV–vis diffuse reflection absorption spectra. The wormlike g-C_3_N_4_ showed better performance for UA degradation in methanol aqueous solution compared with the g-C_3_N_4_ from dicyandiamide under visible light irradiation. The mechanism of enhanced activities was discussed by specific surface area, charge separation efficiency and photoelectric performance. Moreover, the degradation intermediate products of UA after 80 min reaction were proposed based on high resolution mass spectrometry.

## 2. Materials and Methods

### 2.1. Materials

All reagents and UA standard without purification were purchased from Aladdin (Aladdin Bio-Chem Technology Co., LTD, Shanghai, China) and Sigma-Aldrich (Sigma-Aldrich Co. LLC, Shanghai, China). For the analysis of high-performance liquid chromatography and mass spectrometry, acetonitrile and methanol with chromatogram grade were from Sigma-Aldrich. The deionized water was obtained from Milli-Q SP Reagent Water system (Millipore, Bedford, OH, USA).

### 2.2. Synthesis of g-C_3_N_4_ Catalyst

The wormlike g-C_3_N_4_ was prepared from modified pyrolysis method. In brief, 15 g pure dicyandiamide was dissolved in 100 mL water at 60 °C, and the solution was under ultrasonic agitation for 20 min. Then the solution was poured into 5 mL centrifuge tube and dried at 70 °C for 12 h, then the obtained block solid (recrystal) was put into a crucible with a cover. The crucible was heated from 25 °C to 580 °C in 2 h and kept for 4 h. Finally, the as-prepared solid was washed with methanol and water for three times, respectively, and then dried at 80 °C overnight. The wormlike g-C_3_N_4_ (labeled as WCN) was ground into powder in the agate mortar. The pristine g-C_3_N_4_ (labeled as PCN) from dicyandiamide was prepared by direct pyrolysis method according to above method.

### 2.3. Material Characterization

The crystal of catalysts was characterized by X-ray diffraction (XRD) (Bruker AXS, D8, Karlsruhe, Germany) with a scanning range from 10° to 70°. The particle size and morphology of as-prepared samples were characterized by high resolution transmission electron microscopy (FEI Tecnai G2 F30, Hillsboro, OR, USA). X-ray photoelectron spectroscopy analysis of survey, C, N, O elements and valence band spectra were estimated through a Kratos XSAM 800 X-ray photoelectron spectrometer (Kratos Analytical Ltd, Manchester, UK.) with an Mg Kα X-ray source. Diffuse-reflectance UV–vis spectra (DRS) was evaluated by Varian Cary 500 UV–vis-NIR spectrophotometer (Varian medical systems, Palo Alto, CA, USA).

The photoelectrochemical properties of as-prepared catalysts were performed on CHI 760E electrochemical system (Chenhua Instrument Company, Shanghai, China) with three-electrode cell including a working electrode, a mercurous chloride electrode and a Pt counter electrode. For preparation of working electrode, 5 mg of g-C_3_N_4_ was dispersed in 20 µL Nafion solution (5 wt%) and 1 mL absolute ethyl alcohol, and then deposited on a piece of the SnO_2_: F films (FTO). The photocurrent was recorded under a 300 W Xe arc lamp with a filter (*λ* > 420 nm) irradiation in Na_2_SO_4_ aqueous solution (0.2 M). Electrochemical impedance spectroscopy (EIS) was recorded at open circuit potential with the frequency range between 0.1 Hz and 100 kHz, and the magnitude of alternating current voltage was 5 mV.

### 2.4. Photocatalytic Measurement

The photocatalytic activities tests were as follows: 10 mg g-C_3_N_4_ catalyst was dispersed into 95 mL deionized water under 15 min ultrasonic treatment. Next, 5 mL of UA methanol solution (200 μg/mL) was added to above suspension. After that, the suspension was under magnetic stirring for 30 min in the dark to achieve adsorption-desorption equilibrium. Then, the suspension was irradiated by a 300 W Xenon lamp (PLS-SXE 300, Beijing Trusttech Co, Beijing, China) with a filter (*λ* > 420 nm) to carry out photoreaction. The distance between light source and suspension was about 20 cm. The suspension after different irradiation time (20 min, 40 min, 60 min, 80 min) were detected the concentration of UA using HPLC (Shimadzu LC-20A, Kyoto, Japan) equipped with a chromatographic column (Synergi reversed-phase Hydro-C18 column, 5 μm, 250 mm × 4.6 mm, Shimadzu, Kyoto, Japan). The samples were filtrated by a 0.22 μm filter before HPLC detection. Water and methanol 85:15 (*v*/*v*) were used as mobile phase. The flow rate of mobile phase was 1.0 mL/min, and the temperature of column was 40 °C. The detection wavelengths were 220 nm. The injection volume was 20 μL and the total analysis time was 30 min. The total organic carbon (TOC) in suspension was analyzed by TOC analyzer (Shimadzu, TOC-L, Kyoto, Japan).

### 2.5. Degraded Products Identification

The degradation intermediate products of UA were analyzed by HPLC-electrospray ionization mass spectrometry (Thermo Finnigan LTQ XL, Waltham, MA, USA). HPLC (UltiMate 3000 BioRS, Thermo, Waltham, MA, USA) used a Syncronis C18 column (3 μm, 100 × 2.1mm, Thermo, Waltham, MA, USA). The mobile phase was composed of component A (0.1% formic acid) and component B (methanol). The ESI was positive ion mode and the temperature of capillary was at 400 °C. The flow rate of sweep gas, aux gas and sheath gas was at 0 arb, 5 arb, 25 arb, respectively. The nebulizer pressure was 0.45 MPa and the capillary voltage was at 8 V. The tube lens voltage was 100 V. The flow rate was 100 μL/min, and the injection volume of sample was 10 μL. The scanning ranges of MS were from *m*/*z* = 50 to *m*/*z* = 1000.

## 3. Results and Discussions

### 3.1. Crystal Phase and Microstructure

**Figure 2:**
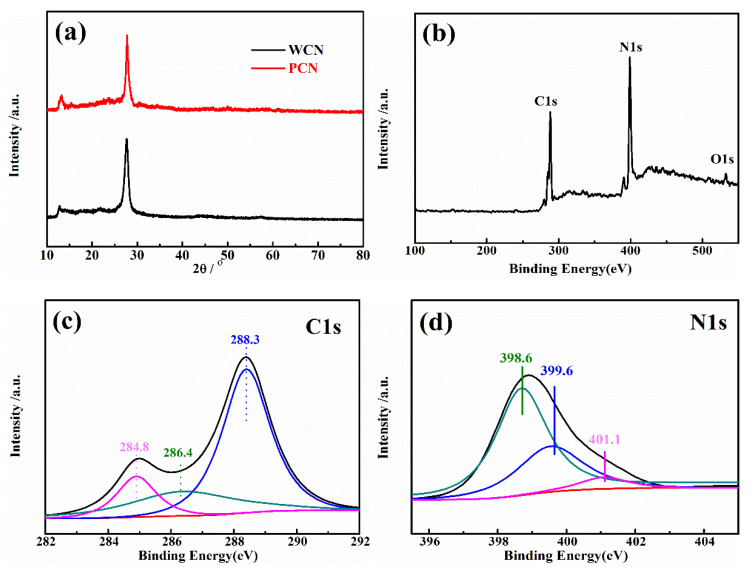
(**a**) XRD pattern of WCN and PCN; (**b**) XPS survey spectra of WCN; high-resolution XPS spectra of C1s (**c**) and N1s (**d**) in WCN.

To analysis the crystal phase of as-prepared catalysts, the X-ray diffraction (XRD) pattern of catalysts were recorded. As presented in Figure 2a, the XRD patterns of the WCN and PCN had two peaks at 27.4° and 13°, which were consistent with (002) and (100) crystal plane [21]. The peak at 27.4° was the characteristic peak of graphitic structure with *d* = 0.326 nm, and the 13° was the peak of in-plane repetitive heptazine frameworks, respectively. There were no obvious other peaks found in two patterns, indicating that the g-C_3_N_4_ was prepared successfully. 

Furthermore, the surface chemical compositions and valence state of C and N element in WCN were estimated through X-ray photoelectron spectroscopy (XPS) characterization. In Figure 2b, it was found that C, H and O elements in the survey XPS spectra. The weak peak at 532.5 eV (O1s) was ascribable to the adsorbed H_2_O or CO_2_. The C1s and N1s spectra were shown in Figure 2c,d. Three peaks at 284.8 eV, 286.4 eV and 288.3 eV were corresponding to graphitic carbon (C–C), C–O and sp^2^-bonded carbon (N–C=N), respectively. The peaks at 398.6 eV and 399.6 eV in N1s spectra were ascribed to sp^2^ hybridized aromatic N bonded to carbon atoms (C=N–C) and the tertiary N bonded to carbon atoms in the form of N–(C)_3_, respectively, and the weaker peak at 401.1 eV was the N–H bond [15,25]. 

**Figure 3:**
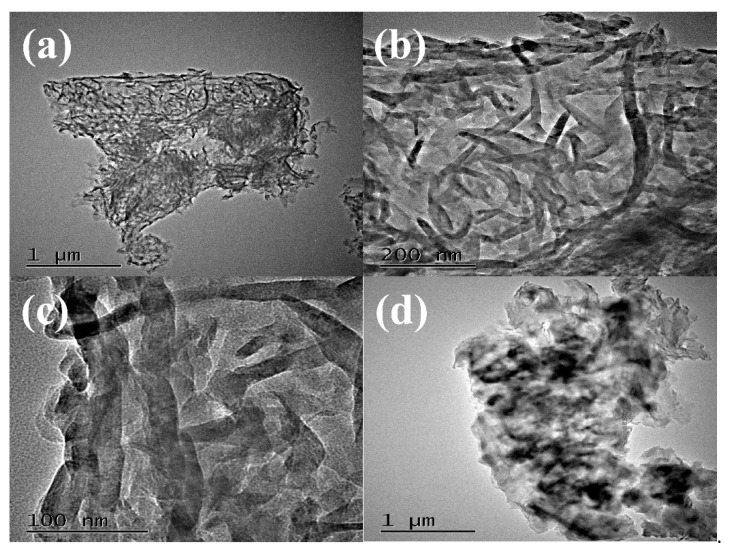
HRTEM images of WCN (**a**–**c**) and HRTEM image of PCN (**d**).

The morphology and microstructure of g-C_3_N_4_ were investigated by high resolution transmission electron microscopy (HRTEM) shown in Figure 3. The images in Figure 3a,b showed that g-C_3_N_4_ was uniform wormlike microstructure. In Figure 3c, the amplifying microscopic image of g-C_3_N_4_ presented the average width of about 10 nm, and the average length of g-C_3_N_4_ was about 100–200 nm. Above HRTEM images indicated that the uniform and regular wormlike g-C_3_N_4_ was successfully prepared through facile pyrolysis method. Moreover, the HRTEM of PCN from dicyandiamide in Figure 3d presented amorphous structure with big blocks. Two catalysts with different microstructures may affect the photocatalytic activities as following. 

### 3.2. Photocatalytic Activity

**Figure 4:**
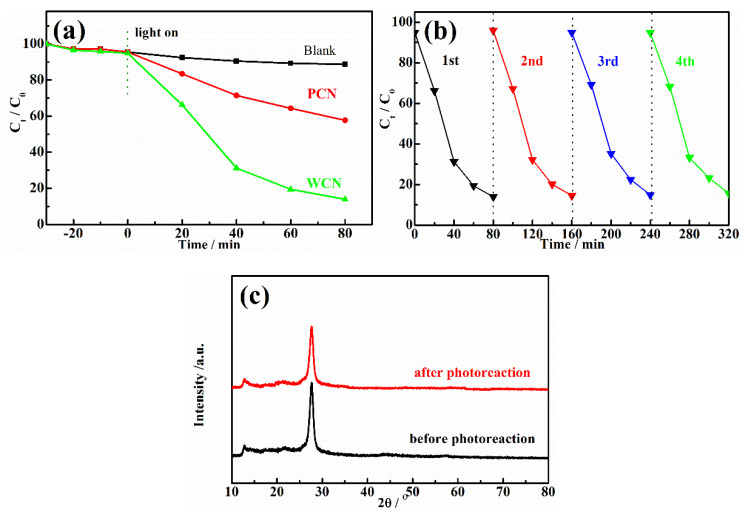
(**a**) Photodegradation of UA over WCN and PCN with different times under visible light irradiation; (**b**) Photocatalytic stability of WCN during four cycles of photocatalytic reaction; (**c**) XRD patterns of WCN before and after four cycles of photocatalytic reaction.

The g-C_3_N_4_ were used as catalysts to estimate the photocatalytic degradation activities of UA under visible light irradiation. The concentration of UA could not decrease under visible light irradiation without catalyst or in the presence of catalyst without visible light irradiation, indicating that the catalyst and visible light were essential for UA degradation. As shown as in Figure 4a, after achieving adsorption-desorption equilibrium for 30 min in the dark, the concentration of UA was reduced slightly. However, it could be found that the reduction of UA in the presence of WCN was higher than that of PCN, indicating that WCN could absorb more UA. After light turned on, it was found that the WCN showed better photocatalytic activity on UA degradation than the PCN. The degradation rate of WCN was 86.1% in 80 min, while it was only 42.3% in the presence of g-C_3_N_4_ from dicyandiamide. To estimate the practical application of catalyst, the stability of WCN was verified by four cycles of experiment as shown as in Figure 4b. It was observed that performance of WCN almost unchanged after the four cycles of test. In addition, there was no obvious changes in XRD patterns after four cycles of reuse experiments (Figure 4c), demonstrating that the WCN was stable and reusable in photoreaction and can be adopted in practical application.

### 3.3. Enhanced Photocatalytic Mechanism of WCN

**Figure 5:**
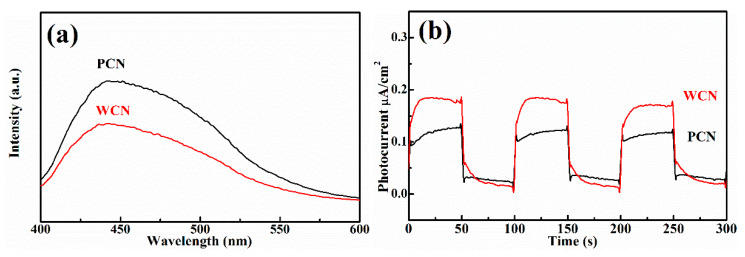

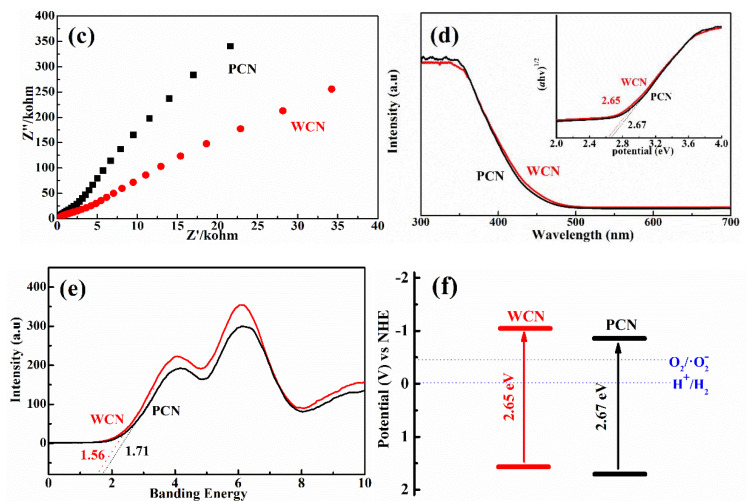
(**a**) Photoluminescence (PL) spectra of WCN and PCN under an excitation wavelength at 360 nm; (**b**) transient photocurrent on-off response of WCN and PCN; (**c**) electrochemical impedance spectroscopy (EIS) of WCN and PCN; (**d**) DRS of WCN and PCN and (*ahv*)^1/2^ as the function of photon energy (*hv*); (**e**) valence band spectra of WCN and PCN; (**f**) band structures of WCN and PCN.

The microstructure of semiconductor nanomaterials affects their photocatalytic properties, which can be characterized through specific surface area, photoluminescence and photoelectric properties. Firstly, it was found that the specific surface area of WCN was 27.2 m^2^ g^−1^, which was larger than g-C_3_N_4_ from PCN with 14.5 m^2^ g^−1^. This indicated the WCN could absorb more UA and provide more active sites to show better activities. In addition, the photoluminescence of two catalysts were investigated, which can reflect the photo-generated charges recombination rate (Figure 5a). It was found that the photoluminescence intensity of WCN was obviously lower than that of g-C_3_N_4_ from dicyandiamide, indicating that the more efficient separation of photo-generated charges on WCN. This might give rise to enhance the photocatalytic performance of WCN for UA degradation compared with PCN.

To further survey the enhancement photocatalytic activities of WCN, the photoelectrochemical abilities of WCN and PCN were compared by evaluating their transient photocurrent and electrochemical impedance spectra (EIS). As shown in Figure 5b, it could be seen that the transient photocurrent intensity of WCN was higher than that of PCN, and two catalysts presented well response during the light on-off recycles. This indicated that WCN could generate higher photocurrent under visible light irradiation. The EIS of WCN also showed the smaller diameter of the Nyquist plots compared with that of PCN (Figure 5c), demonstrating the better photogenerated charges transfer in the WCN. Therefore, the higher photocurrent and better photogenerated charges transfer of WCN were benefit to the photocatalytic activities, which may be ascribe to the uniform microstructure of WCN and the shorter charges transfer distance from interior to exterior on the surface of wormlike microstructures [24]. As shown in Figure 5d, it was found that the WCN and PCN had the visible absorption edges at about 460 nm, and the bandgaps were 2.65 and 2.68 eV, respectively, according to the formula (*ahv* = A (*hv*−*E*_g_)*^n^*^/2^, where *A*, *a*, *hv*, *E*_g_ and *n* are the constant value, absorption value, light energy, bandgap value and 1 of *n* value for g-C_3_N_4_, respectively) [14,31]. The valence band of WCN and PCN were evaluated to 1.56 V and 1.71 V as shown in Figure 5e. The band structures of catalysts were presented in Figure 5f after band structure calculation. It was found that WCN showed the more negative conduction band of −1.09 V than PCN of −0.97 V, demonstrating that WCN had stronger thermodynamic driving force for photo-reduction that may promote the degradation of UA.

### 3.4. Degradation Intermediate Products 

**Figure 6:**
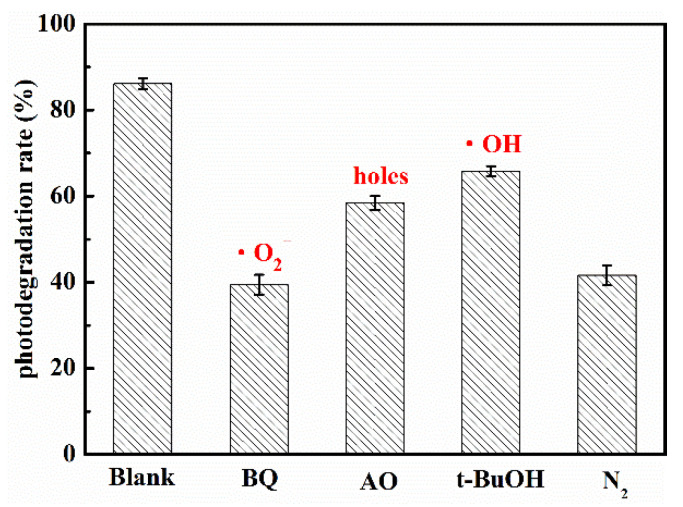
Trapping tests of the active species for the photocatalytic degradation of UA.

The photocatalytic reaction mainly depended on the kinds and quantity of the active radicals. When the energy of light was higher than bandgap of semiconductor, the electrons was excited and transferred from valence band to conduction band, while holes was left in the valence band. Then, the electrons on conduction band reacted with dissolved O_2_ in water to form superoxide radical anions (•O_2_^−^), which had strong reducing capacity. The holes might oxidize the OH^−^ to hydroxyl radicals (•OH). Finally, the active groups such as electrons, holes, •O_2_^−^ and •OH and so on reacted with the target organic molecules, and the target organic molecules were degraded steps by steps to final product such as CO_2_ and H_2_O. For investigating the role of active radicals in photodegradation of UA in the presence of WCN, the different scavenger reagents trapping measurement were carried out. The *tert*-butanol (*t*-BuOH), ammonium oxalate (AO) and 1,4-benzoquinone (BQ) were as •OH, holes and •O_2_^−^ scavengers, respectively. As shown in Figure 6, the degradation rate decreased obviously when the BQ added in solution, indicating the •O_2_^−^ was the main active group for UA degradation. The control test of N_2_ purging also confirmed the important role of •O_2_^−^ during the photocatalytic reaction. However, when the AO and BQ added in suspension, there were slight reduction compared with without scavenger reagents (Blank). This may be ascribed to the valence band edges of g-C_3_N_4_ were lower than that of H_2_O/•OH, +2.40 V vs NHE (normal hydrogen electrode) that cannot directly oxidize the H_2_O or OH^−^ to •OH. From previous reports [32,33], the small number of •OH may be indirectly from the following reactions of •O_2_^−^ in water during photoreaction (O_2_ + e− → •O_2_^−^, •O_2_^−^ + e^−^ + 2H^+^ → H_2_O_2_, H_2_O_2_ + e^−^ → •OH + OH^−^). 

**Figure 7:**
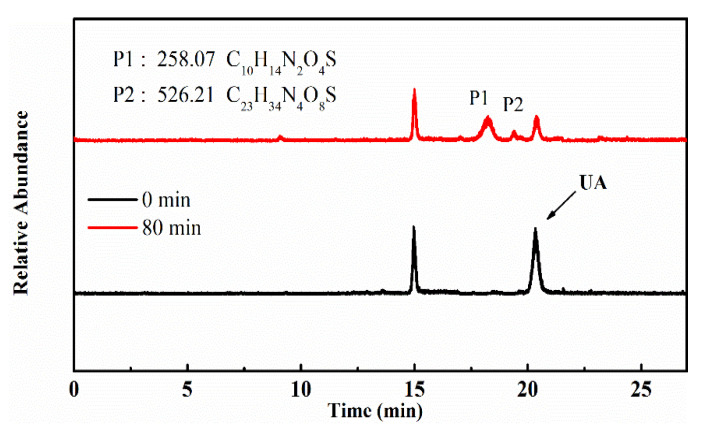
TIC of the UA before and after 80 min photoreaction, and chemical formula of P1, P2.

**Figure 8:**
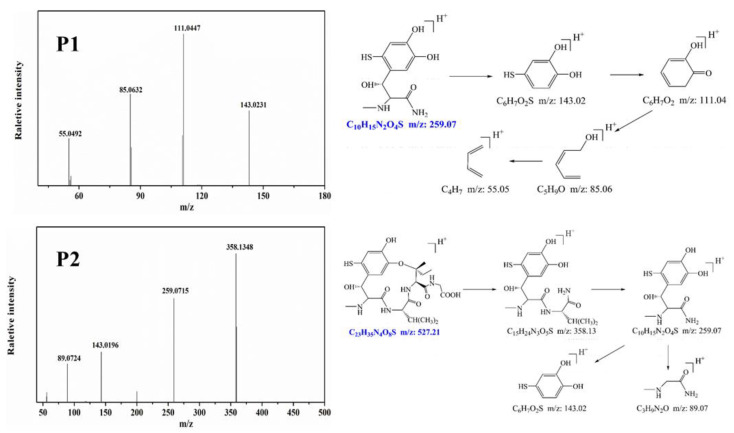
MS/MS spectra and proposed fragmentation of intermediates products or of UA (P1 and P2).

**Figure 9:**
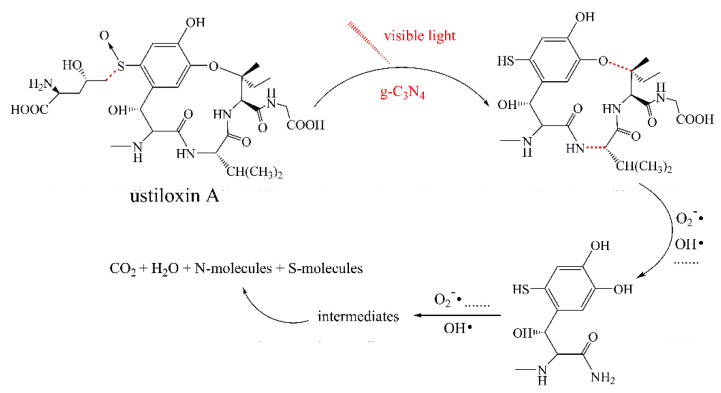
Possible degradation pathway of UA over g-C_3_N_4_ under visible light irradiation.

**Figure 10:**
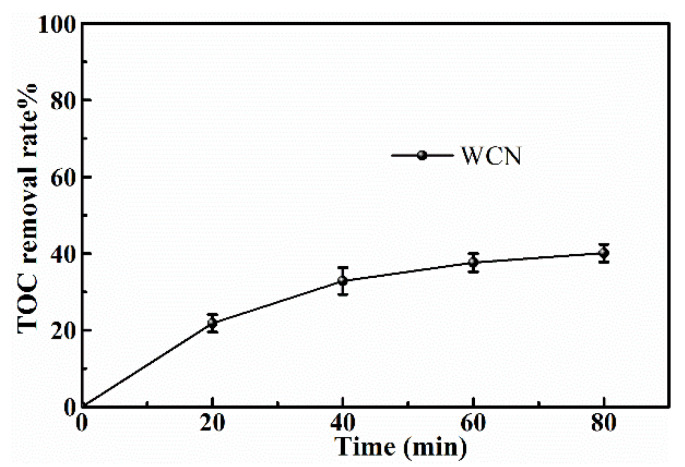
TOC removal plots of UA over WCN under visible light irradiation.

To identify the photocatalytic degradation intermediates of UA, the products after photocatalytic treatment for 80 min were recorded by HPLC-MS as shown in Figure 7. The peak at about 15 min was solvent peak. After 80 min photoreaction, there are two intermediate products observed in the Total Ion Chromatograms (TIC) of the photocatalysis treated sample. The possible chemical formula of intermediate products (P1: C_10_H_14_N_2_O_4_S and P2: C_23_H_34_N_4_O_8_S) were deduced according to software computational deduction. In addition, the possible structure of two intermediate products were proposed based on the MS/MS spectra and fragmentation formation of P1 and P2 shown in Figure 8. These two intermediate products generated from active radical reaction with the UA molecular during complex redox reaction gradually, and the possible pathway of UA degradation was as shown in Figure 9. Firstly, the active groups such as •OH, •O_2_^−^ etc. reacted with the S–C band in the right side, and then loosed the adjacent group to form P2. The 13-membered cyclic core structure was opened after the breakage reactions of C–O and C–N band to generate the P1 with more stable structure. Then the P1 might be gradual degradation to form smaller molecules, and even the final products such as CO_2_, H_2_O, nitrogenous and sulphureous molecules. To confirm the proposed pathway of UA, the TOC in the suspension was recorded as the visible light irradiation in the presence of WCN (Figure 10). It can be found that TOC in solution decreased, illustrating that the UA toxic might be degraded thoroughly to CO_2_, H_2_O etc. From above results and discussions, it can be concluded that UA as a cyclopeptide toxin, the main toxic structure of 13-membered cyclopeptide was destroyed after photoreaction, indicating the WCN could be as an efficient and promising visible light catalyst for UA degradation and its toxicity mitigation.

## 4. Conclusions

In conclusion, a kind of wormlike g-C_3_N_4_ catalyst with visible light-responsive was successfully synthesized from modified dicyandiamide precursor through a facile pyrolysis synthesis method. The WCN showed better activity and stability for degradation of UA in methanol aqueous solution compared with that of PCN under visible light irradiation, which was ascribed to that the uniform microstructure, larger specific surface area, efficient photo-generated charges separation and transformation that can provide more active sites and generate more active groups on the surface of catalyst to degrade the UA. Moreover, two intermediate degradation products and the possible pathway were proposed through HPLC-MS/MS after 80 min photoreaction. This work provided an efficient, green and promising way to reduce UA contamination for future study.
